# Supplementary material for: Collaborative intelligence in AI: Evaluating the performance of a council of AIs on the USMLE
Source: PLOS Digit Health. 2025 Oct 9;4(10):e0000787. doi: 10.1371/journal.pdig.0000787 (PMC12510544; doi:10.1371/journal.pdig.0000787)
Supplement: S2 File — (ZIP) [file pdig.0000787.s002.zip › S2 Data Raw data files containing unprocessed question inputs and LLM outputs including transcripts of deliberations/STEP3_Question-126-1_Type-MC-NJ.docx]

**Facilitator** **[Round 1]:** A 25-year-old woman comes to the office because of a 24-hour history of right-sided facial weakness. Two days ago she developed mild aching behind the right ear, which resolved spontaneously. This morning, however, the entire right side of her face was drooping and she was unable to close her right eye. When she tried to drink coffee this morning, the liquid ran out the right side of her mouth. She has been otherwise healthy and she takes no medications. Vital signs are normal. Examination of the ears and oral mucosa shows no abnormalities. Cranial nerve examination shows moderate weakness of the right orbicularis oculi and oris, frontalis, buccinator, and platysma muscles. Sound is perceived as louder in the right ear. The remainder of the neurologic examination shows no abnormalities. Which of the following is the most accurate statement regarding this patient's prognosis for neurologic recovery? (A) Prognosis cannot be determined without results of cerebral angiography (B) Prognosis cannot be determined without results of MRI of the brain and cerebrospinal fluid analysis (C) She has an 80% chance of complete recovery within the next 12 months without treatment (D) With oral corticosteroid therapy she has a 20% chance of complete recovery within the next 12 months (E) It is unlikely that the patient will achieve functional recovery from this condition regardless of therapy

**AI_D:** RESTATING THE QUESTION: The question is asking what the most likely outcome for this 25-year-old woman, who appears to be suffering from some form of neurological defect causing facial weakness, is in terms of recovery. Specifically, it wants to know if her prognosis can be determined and if so, what that prognosis might be. PLANNING: The assumptions identified include: 1) The symptoms point to a specific neurological condition 2) The specific neurological condition can be diagnosed with specific investigations or managed with specific treatments. By questioning these assumptions, the alternate assumption would be that the patient's symptoms don't fit a specific neurological condition which can conclusively be diagnosed or managed predictably. The clinically prioritized assumption to pursue is the likelihood of a specific condition, based on the provided symptom analysis. CLINICAL REASONING: The patient presents with symptoms of sudden unilateral facial weakness which includes inability to close the eye, drooping of the face, and spilling of liquids from the corner of the mouth. These are classic signs of Bell's palsy, a condition involving inflammation of the facial nerve that typically results in a rapid onset of weakness on one side of the face, often over the course of a few hours. The cause is often unknown, but it can be associated with viral infections. Interestingly, she also exhibits hyperacusis (sound perceived as louder in the right ear), which is a common but often under-recognized symptom of Bell's palsy, as acute facial nerve inflammation can lead to increased stimulation of the stapedius muscle in the middle ear. CONCLUDING: Given these clinical features that seem consistent with Bell's palsy and our understanding of the typical course of this disease, predicting the prognosis for this patient can be more straightforward than certain other neurological conditions. RESPONSE: The best answer would be (C) She has an 80% chance of complete recovery within the next 12 months without treatment. Bell's palsy indeed has a favorable prognosis with about 70-80% of patients recovering fully within three to six months, even without treatment. And this prognosis goes up to 85-90% when treatment with oral corticosteroids, such as prednisone, is initiated within 72 hours of symptom onset. Thus, the prognosis could even potentially be better if she was given corticosteroid treatment, not worse as suggested in option (D).

**AI_A:** RESTATING THE QUESTION: The 25-year-old woman in this case has experienced a sudden onset of weakness in her facial muscles, particularly on the right side. This has resulted in drooping of her right facial side, inability to close her right eye, and problems with oral intake. The examination has revealed no additional abnormal findings other than weakness of various facial muscles and hypersensitivity to sound in the right ear. This suggests an ailment of the facial nerve, which supplies these muscles. The query here is about the prognosis of this putative facial nerve disorder and we're presented with five potential responses. PLANNING: Each of the assumptions here relates to a possible outcome for her condition, with explicit or implicit assumptions about its etiology and treatment. For instance, (A) and (B) suppose that her prognosis can't be determined without more advanced neuroimaging (cerebral angiography or MRI plus CSF analysis) – suggesting that these tests might unveil an underlining issue that's influencing her prognosis. In contrast, (C) and (D) presume that the prognosis hinges on either natural recovery or the effectiveness of corticosteroid therapy. Option (E) implies the condition is likely to be irreversible. CLINICAL REASONING: This is a typical case of Bell's palsy, an idiopathic condition characterized by acute peripheral facial nerve palsy. The facial nerve innervates the muscles responsible for facial expression, hence the inability to close the eye or control the mouth on the affected side. Another sign is hyperacusis (increased hearing sensitivity), caused by loss of stapedius muscle innervation (which dampens sound). Bell's palsy is a diagnosis of exclusion, made when no other cause of facial nerve palsy is identified on clinical grounds or via appropriate investigations, such as brain MRI in patients with atypical features or those without improvement in symptoms. The disease usually has a benign course, with many patients recovering spontaneously. CONCLUDING: Given the typical presentation, the diagnosis of Bell's palsy is probable and the nature of the disease means most patients will see considerable improvement or complete recovery even without treatment. Corticosteroid therapy can further improve the chances of recovery. Considering this, the options suggesting additional neuroimaging to determine a prognosis or implying negligible chance of recovery don't align with what is generally known about the disease. RESPONSE: Therefore, I'd suggest that (C) 'She has an 80% chance of complete recovery within the next 12 months without treatment' is the most accurate reflection of this woman's prognosis, given the usual course of Bell's palsy.

**AI_E:** 1) RESTATING THE QUESTION: The patient is a 25-year-old woman who appears to be generally healthy. However, she's been experiencing right-sided facial weakness and earache from the past 24 hours. Her right side of the face was drooping this morning, and she was not able to close her right eye or drink without spilling. Cranial nerve examination revealed that certain facial muscles, such as the right orbicularis oculi and oris, frontalis, buccinator, and platysma, are moderately weak. It is also noted that sounds are louder in her right ear. The question is about the prognosis of her neurological recovery. 2) PLANNING: The symptoms suggest the presence of a disorder involving the facial nerve, possibly Bell's palsy. Prognosis for this condition generally depends on the cause and the treatment initiated. The assumptions in this case are that the patient's condition is due to an underlying disease that can be detected through additional tests or that it is simply a transient issue that will resolve on its own or with steroid therapy. 3) CLINICAL REASONING: Apart from weakness of some of the facial muscles and perceived loudness in the right ear, there are no other abnormal neurological signs. This coupled with the sudden onset of symptoms, suggests that this might indeed be Bell's palsy caused by inflammation of the facial nerve, not due to an identifiable underlying disease that could be seen on an MRI or cerebral angiography. 4) CONCLUDING: Based on this line of clinical reasoning, we can rule out (A) and (B) as unnecessary, given the absence of other neurological signs. Considering that Bell's palsy is a disease with a generally favorable prognosis, especially in the absence of other comorbidities or predisposing conditions, it is safe to rule out (E) as well. 5) RESPONSE: Between options (C) and (D), given that Bell's palsy is often self-limiting and that the majority of patients recover with or without treatment, and bearing in mind that the patient is young and otherwise healthy, option (C) "She has an 80% chance of complete recovery within the next 12 months without treatment" is the most accurate statement regarding her prognosis. It is, however, important to note that more studies do suggest better outcomes with early treatment primarily using corticosteroids.

**AI_B:** The question is asking us to consider the various potential prognoses of a 25-year-old woman who presents with right-sided facial weakness, inability to close her right eye, and difficulty preventing liquids from escaping the right side of her mouth. These symptoms, along with a history of mild aching behind the ear which resolved spontaneously, are typically congruent with a condition known as Bell's Palsy. Bell's palsy is a neuropathy (nerve disorder) that affects the facial nerve, also known as the 7th cranial nerve, causing sudden, unilateral (one-sided), temporary facial paralysis. Hypersensitivity to sound in one ear, also known as hyperacusis, is also a common symptom, which aligns with the patient's presentation. It's important to first take into consideration the listed possible prognoses and assess their individual merits: (A) needing a cerebral angiography, (B) requiring an MRI and cerebrospinal fluid analysis, (C) her being able to recover fully within 12 months without treatment, (D) a 20% chance of complete recovery with oral corticosteroid therapy within 12 months, (E) and the improbability of her achieving functional recovery regardless of therapy. Given the clinical presentation, the most probable diagnosis is Bell's Palsy, which typically has a good prognosis with or without treatment. Full recovery generally occurs within three to six months in most cases. Examining patterns A and B, while imaging tests may aid in distinguishing a central cause like a stroke from a peripheral cause like Bell’s palsy, the good prognosis of Bell's Palsy often allows for a diagnosis based purely on clinical presentation, invalidating the need for extensive imaging in this particular case. The focus then shifts to patterns C, D, and E, with the likelihood of recovery being a significant factor. Critical evaluation of the clinical case helps us connect various medical domains: from the pathology and pathogenesis of Bell's Palsy, a peripheral cranial neuropathy, to its symptomatology (the domain of internal medicine) and treatment options (the domain of pharmacology). With a judicious mix of these domains, we can critically assess the above-mentioned possibilities. Considering the diagnosis is Bell's Palsy and barring unforeseen complications or pre-existing neurological conditions, it's most probable that the patient's prognosis aligns with pattern C – an 80% chance of complete recovery within the next 12 months without treatment. Bell's Palsy is self-limiting and most patients recover fully without any specific therapy. It's worth noting that corticosteroids may help speed up recovery in some cases, but they are not necessary for recovery to occur. This challenges pattern D, which implies a low chance of recovery even with corticosteroid treatment. Pattern E underestimates the prognosis of most patients with Bell's Palsy, who usually achieve functional recovery. In conclusion, provided this presentation of unilateral facial weakness with hyperacusis is indeed Bell's Palsy, the patient has a good prognosis, specifically an 80 percent chance of complete recovery within the next 12 months, even without any specific treatment. Thus, pattern C appears to provide the most accurate prognosis.

**AI_C:** Restating the question, we are asked to determine the prognosis for a 25-year-old female patient presenting with recent unilateral facial weakness, preceded by a mild aching sensation behind the ear that resolved quickly. Notably, she's experiencing symptoms compatible with Bell's palsy, a condition often distinguished by sudden onset facial paralysis. Crucially, no abnormalities were detected during her general and neurological examinations, and sound perception is unusually heightened in her affected ear. A few preliminary assumptions can be made to guide our clinical reasoning. A primary assumption, based on the information given, is that the patient might have Bell's palsy. This assumption springs from her sudden unilateral facial weakness, the absence of other significant neurological abnormalities, and hyperacusis (increased sound sensitivity), which is a distinctive feature of Bell's palsy. An alternate assumption could be that this patient's condition is a symptom of a more severe neurological disorder that requires further investigation, such as an MRI or cerebral angiography - we would need to consider the potential impact of not diagnosing a more severe ailment promptly. However, based on the clinical presentation and the general health of the patient, Bell's palsy seems to be the more plausible assumption. Beginning with this more clinically prioritized assumption, we can unpack the patient's prognosis. Bell's palsy is generally understood as a condition caused by inflammation of the Facial Nerve (Cranial Nerve VII) - this is responsible for her moderate weakness of various muscles on her face. This inflammation can cause symptoms ranging from mild facial weakness to complete paralysis on one side of the face, like in our patient. Considering the prognosis of Bell's palsy, commonly, the majority of patients experience a good prognosis, with significant improvement or complete resolution often seen within three to six months, even in the absence of treatment. However, early treatment with oral corticosteroids has been shown to increase the chance of complete recovery. Docking from this analysis, the answer likely lies between options C and D - she has a high chance of complete recovery either with or without treatment. However, given that the chance of recovery in Bell's palsy without treatment is generally high, but is even higher (nearing 100%) with oral corticosteroid therapy, none of these options appears completely appropriate. Turning to the response, it seems that none of the options given explicitly delineates the patient's prognosis as per our analysis. However, option (C) appears closest in indicating a high likelihood of complete recovery within the next 12 months even without treatment. Nevertheless, it's important to note in practice patients with Bell's palsy are usually treated with corticosteroids to maximize the chance of full recovery, not reflected in the answer options.

**Facilitator** The consensus among the AI responses is that the 25-year-old woman's symptoms, including sudden onset of right-sided facial weakness, inability to close her right eye, and hypersensitivity to sound in the right ear, are indicative of Bell's palsy. This condition is characterized by inflammation of the facial nerve, leading to unilateral facial paralysis. Bell's palsy is typically a diagnosis of exclusion, made when no other cause of facial nerve palsy is identified through clinical examination or appropriate investigations. The prognosis for Bell's palsy is generally favorable, with a significant number of patients experiencing considerable improvement or complete recovery within three to six months, even without treatment. Early treatment with oral corticosteroids can further enhance the chances of complete recovery. Therefore, the need for advanced neuroimaging such as cerebral angiography or MRI, as suggested in options (A) and (B), is not aligned with the typical course of Bell's palsy. Similarly, option (E), which implies a negligible chance of recovery, does not reflect the usual prognosis of this condition. Given these considerations, the consensus is that option (C), stating that the patient has an 80% chance of complete recovery within the next 12 months without treatment, provides the most accurate prognosis. However, it's important to note that in practice, patients with Bell's palsy are often treated with corticosteroids to maximize the chance of full recovery, a detail not explicitly reflected in the answer options.
